# Transforming optical fibers from passive transmitters to multimodal sensory platforms

**DOI:** 10.1093/nsr/nwag386

**Published:** 2026-06-24

**Authors:** Yonggang Huang

**Affiliations:** McCormick School of Engineering, Northwestern University, USA

Optical fibers underpin modern communication infrastructure and increasingly serve as platforms for distributed sensing. Conventional fiber sensing technologies based on Rayleigh, Brillouin and Raman scattering enable large-scale monitoring of temperature and strain [1–3]. Such approaches fundamentally rely on perturbations of guided optical modes, which restrict sensing modalities and functional integration. Realizing multimodal perception and active signal processing directly on fine fibers remain a long-standing challenge.

In a recent paper published in *National Science Review* [4], Wang and co-workers report an electrical-sensing–optical-transmission fiber sensor (ESOT FiSensor) that transforms optical fibers from passive waveguides to a multifunctional sensory platform. Instead of engineering light–matter interaction within the fiber core, the authors integrate hybrid electronic–photonic circuits directly onto the fiber surface through conformal electrohydrodynamic printing (e-Printing).

**Figure 1.**
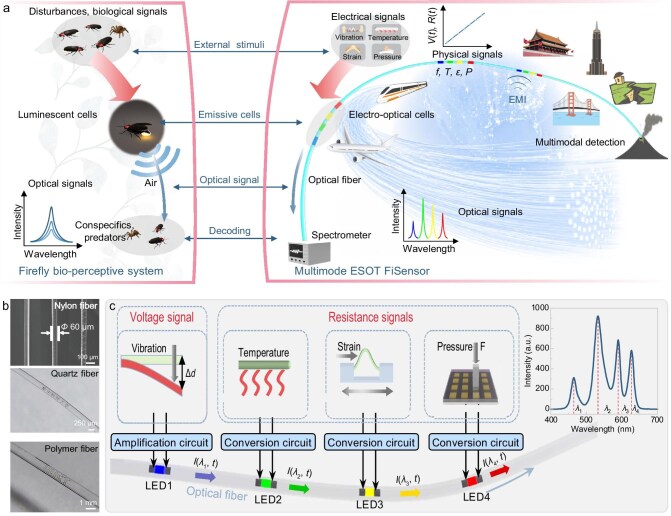
The architecture and application of the ESOT FiSensor for multimodal physical signal sensing and transmission. (a) The signal-sensing process and application scenarios of the multimodal ESOT FiSensor. (b) Different types of printed lines on the surface of a nylon fiber with a diameter of 60 μm, and complex printed patterns on quartz fiber and polymer fiber surfaces. (c) Schematic illustration of the ESOT sensing process for multiple physical stimulations including vibration, pressure, temperature, and strain.

In general, direct printing on cylindrical substrates with diameters down to tens of micrometers poses severe challenges for conventional microfabrication due to large surface curvature and unstable jet–substrate interactions. By adopting an advanced e-Printing strategy, the authors achieve stable jetting and continuous patterning on fine fiber surfaces without requiring grounded electrodes [5]. This polar-assisted strategy overcomes the long-standing bottlenecks of jet instability and geometric distortion inherent to conventional microfabrication on high-curvature substrates. The e-Printing enables conformal printing of patterns with line feature sizes down to ~260 nm on fibers with a diameter of ~100 μm, representing one of the highest printing resolutions achieved on such highly curved substrates. This enables the direct integration of sensing elements, conversion circuits, and microscale light sources onto the fiber body (Fig. 1). In contrast to previous fiber-based multimodal sensors that rely on optical interference or structural modulation (such as fiber Bragg gratings or interferometers) [6,7], which fundamentally suffer from signal cross-talk and require bulky, external spectrometers or active light sources, this approach externalizes functionality to the fiber surface, greatly expanding integration capability. By combining the versatile physics of electronic sensing with advanced optical transmission, the ESOT FiSensor circumvents the complex demodulation and intrinsic coupling limits of traditional fiber sensors, offering a scalable solution for infrastructure-level networks.

The ESOT architecture converts the measured electrical signals from attached sensing units into wavelength-encoded optical outputs. It also establishes a new sensing-and-transmission strategy that allows virtually arbitrary electrical sensors to be integrated onto fiber surfaces for long-distance optical transmission. Multiple stimulations, including vibration, temperature, strain and pressure, are independently detected and multiplexed through wavelength-division transmission along a single fiber over 50 m, with low inter-channel crosstalk. This electro-optical encoding strategy decouples sensing physics from intrinsic optical scattering mechanisms, overcoming a key limitation of conventional distributed fiber sensors. It fully combines the functional versatility of electrical sensing with the advantages of optical transmission, including long-distance signal delivery, distributed multiplexing capability and strong resistance to electromagnetic interference. The robustness and scalability of the system are demonstrated through distributed multimodal monitoring in realistic scenarios. These include simultaneous vibration–temperature detection on moving platforms, spatial temperature detecting on aircraft wings, and gesture recognition for human–machine interaction with classification accuracy exceeding 98%. The ESOT FiSensor can also maintain stable performance under cyclic bending.

Conceptually, this study represents a paradigm shift in fiber technology. Rather than modifying internal optical modes, functionality is built externally via scalable conformal printing. Such an approach opens the possibility of upgrading existing fiber networks into distributed, multimodal sensory skins for aerospace systems, intelligent transportation, subsea monitoring and robotic systems. By bridging flexible electronics and guided-wave photonics at the fiber level, this study provides a promising route toward infrastructure-scale intelligent sensing platforms. Together with recent advances in fiber-integrated computing, fiber bioelectronics and mechanically intelligent robotic systems [8–10], this study further highlights the emerging trend of embedding sensing, computation and actuation directly into fiber-like architectures, including fiber chips, bioelectronic fibers and intelligent robotic fibers.
